# Relationship between long working hours and smoking behaviors: Evidence from population-based cohort studies in Korea

**DOI:** 10.5271/sjweh.4147

**Published:** 2024-05-01

**Authors:** Seong-Uk Baek, Myeong-Hun Lim, Taeyeon Kim, Yu-Min Lee, Jong-Uk Won, Jin-Ha Yoon

**Affiliations:** 1 Department of Occupational and Environmental Medicine, Severance Hospital, Yonsei University College of Medicine, Seoul, Korea.; 2 The Institute for Occupational Health, Yonsei University College of Medicine, Seoul, Korea.; 3 Graduate School, Yonsei University College of Medicine, Seoul, Korea.; 4 Department of Preventive Medicine, Yonsei University College of Medicine, Seoul, Korea.

**Keywords:** health behavior, health-related behavior, lifestyle, nicotine, overtime work, overwork, tobacco, working time

## Abstract

**Objectives:**

Long working hours and overwork are growing public health concerns in the Western-Pacific region. We explored the relationship between working hours and smoking behaviors of Korean workers.

**Methods:**

This study included 284 782 observations (50 508 workers) from four nationwide cohort studies in Korea. Using generalized estimating equations, we estimated the associations of working hours with current smoking status, smoking initiation, and smoking cessation within each cohort. Cohort-specific estimates were combined through random-effect meta-analysis. Effect sizes were presented as odds ratios (OR) and 95 confidence intervals (CI).

**Results:**

The overall smoking prevalence was 26.8% within the cohorts. The adjusted OR (95% CI) of the association between working hours and current smoking were 1.01 (0.94–1.08) for <35 hours/week, 1.04 (1.01–1.09) for 41–48 hours/week, 1.06 (1.01–1.10) for 49–54 hours/week, and 1.07 (1.04–1.10) for ≥55 hours/week compared with 35–40 hours/week. The adjusted OR (95% CI) of the association between working hours and smoking cessation in the follow-up were 0.93 (0.85–1.02) for <35 hours/week, 0.89 (0.83–0.96) for 41–48 hours/week, 0.87 (0.81–0.95) for 48–54 hours/week, and 0.91 (0.85–0.98) for ≥55 hours/week compared with 35–40 hours/week. No clear associations were observed between working hours and smoking initiation.

**Conclusion:**

Long working hours are associated with high current smoking risk and reduced likelihood of smoking cessation among Korean workers. Policy interventions are required to promote smoking cessation and reduce excess overwork for individuals experiencing long working hours.

In countries in the Western-Pacific regions, long working hours are a major public health concern. A recent study conducted by the World Health Organization (WHO) and International Labor Organization (ILO) estimated that the prevalence of long working hours, as defined as ≥55 hours/week, has been increasing globally, and approximately 488 million people are subjected to long working hours (1). Furthermore, recent changes in economic structure – such as the advent of industry 4.0 and gig economy – have the potential to accelerate this trend. The health burdens of long working hours are worrisome considering that they cause ischemic heart disease and stroke (2, 3) and lead to dealth – approximately 745 000 in 2016 according to the WHO/ILO reports (1). Korea has ranked as a country with high annual working hour, and overwork-related death, so-called “karoshi,” which has been a major concern among public health researchers and policymakers (4, 5).

Smoking is a well-recognized public health risk factor associated with the development of various acute and chronic disorders, including cardiovascular diseases. Despite a gradual decline in smoking prevalence over the past decades in Korea, it continues to be a major socioeconomic burden (6). Smoking within the working population is of particular concern in occupational health; smoking is considered to play a mediating role in the link between long working hours and adverse health outcomes, including ischemic heart disease and stroke (3, 7–9). Therefore, exploring the association between long working hours and smoking behavior can provide novel insights into understanding the mechanisms underlying the health effects of long working hours and identifying areas where policy interventions may be required.

Previous studies have investigated the association between long working hours and smoking habits (10–14); however, the results have been mixed. For instance, Angrave et al (10) have found that working ≥50 hours/week is associated with increased smoking risk among the UK and Australian workers. Similarly, previous cross-sectional studies in Korea have found that long working hours are associated with reduced intention to quit smoking (11) and high likelihood of current smoking (12–14), maintaining that workers may compensate for overwork-related job stress by smoking. Conversely, other studies observed no clear associations between working hours and smoking (15–18). For instance, Jang et al (18) maintained in their study that the high smoking prevalence among workers subjected to long working hours may be attributed to disparities in socio-economic status.

The main limitation in the existing body of literature is that most studies investigating the relationship between working hours and smoking behaviors of workers were based on a cross-sectional design. Therefore, only little is known about how working hours affect workers’ willingness to start or quit smoking over time. Thus, this study aimed to explore the association of working hours with current smoking, smoking initiation, and smoking cessation among workers, based on the nationwide cohort studies in Korea.

## Methods

### Datasets and study populations

We used the following nationwide, population-based panel databases that include information on working hours and smoking behaviors within the Korean population: the Korean Labor and Income Panel Study (KLIPS), the Korea Welfare Panel Study (KWPS), the Korea Health Panel Survey (KHPS), and the Korean Longitudinal Study of Aging (KLoSA).

A longitudinal survey conducted annually by the Korea Labor Institute, KLIPS collects data on individuals aged ≥15 years living in Korea. Because smoking status data have been collected since 2005, we included the survey participants from 2005 to 2021. Conducted annually by the Korea Institute for Health, KWPS is a longitudinal survey targeting the general population living in Korea. We included the survey participants from 2008 to 2022 as smoking status data were collected from 2008. Conducted annually by the Korea Institute for Health and Social Affairs from 2008 to 2018, KHPS was also a longitudinal survey targeting the general population living in Korea. However, data on working hours were collected only for paid workers from 2011 to 2014. Another longitudinal survey, KLOSA targeted middle-aged and older Korean adults aged ≥45 and was conducted by the Korea Employment Information Service biannually from 2006 to 2020. Survey participants from 2006 (Wave 1) to 2020 (Wave 8) were included for analysis. Each dataset includes a nationally representative sample of the targeted Korean population, using a stratified cluster sampling method that used geographic regions and households in each area as sampling units. Trained interviewers, employed by each survey-conducting institution, conducted face-to-face interviews to gather information. Further details – including the number of survey participants, observation periods, and survey questionnaires – are provided in the supplementary material (www.sjweh.fi/article/4147), table S1.

The flowchart of the selection process of study participants is presented in supplementary figure S1. First, we excluded the observation in which the survey participants did not participate in economic activities, thus including only workers. Next, we excluded the observations containing any missing values; approximately 2.0% of the total observations were dropped. Thus, 284 782 observations (50 508 individuals) were used to illustrate the sample’s descriptive statistics and investigate the cross-sectional relationship between working hours and concurrent smoking status. Subsequently, for the analysis of smoking initiation, we included 174 650 observations in which the survey participants reported not smoking and 64 255 observations in which the participants reported smoking.

### Ethics statement

The Institutional Review Board of Severance Hospital reviewed and approved this study (No. 4–2023–1184).

### Variables

All variables were repeatedly measured over time for each survey participant. The main exposure variable was working hours per week, which was self-reported. In line with previous WHO/ILO studies that examined the health effect of long working hours (1–3, 19), working hour per week was categorized as <35, 35–40, 41–48, 49–54, and ≥55 hours. Those working standard hours (35–40 hours/week) were considered the reference group.

Regarding outcome variables, self-reported status of cigarette smoking was collected. Current smoking, smoking initiation, and smoking cessation were the outcomes of interests. First, for each survey wave *t*, those who reported that they were currently smoking in wave *t* were defined as current smokers. Second, for each survey wave *t*, those who reported they did not smoke in wave *t*, but did in the following wave (wave *t+1*) were defined as having initiated smoking. Third, for each survey wave *t*, those who reported that they smoked in wave *t*, but did not in the following wave (wave *t+1*) were defined as having ceased smoking. In smoking initiation and cessation analyses, observations with missing values on the smoking status of the following wave were excluded from the analyses. The survey questions regarding cigarette smoking did not distinguish between conventional cigarette and e-cigarette use in all surveys, encompassing both types of smoking.

The selection of confounders was based on previous studies that examined the potential effect of working hours and smoking behaviors (10–14). Gender was adjusted. Age was categorized into <30, 30–39, 40–49, 50–59, and ≥60 years. Education level was categorized as having completed middle school or below, high school, and college or above. Income level was categorized according to the quartile values of household income for each survey year and cohort. Marital status was categorized into married, unmarried, and other (widowed, separated, divorced). Occupation type was categorized into blue collar (employee), blue collar (employer), white collar (employee), and white collar (employer) based on the Korean Standard Classification of Occupation. Additionally, we adjusted year-specific effects by including dummy variables corresponding to each survey year into regression models. All cofounders were collected in each survey and treated as time-varying covariates.

### Statistical analysis

We employed a two-step meta-analysis, in which estimates were obtained from each dataset at the first stage, and cohort-specific estimates were combined through random-effect meta-analysis. For each dataset, we employed a generalized estimating equation (GEE) considering that our primary objective was to estimate the population-averaged effect of long working hours on smoking behaviors (20). First, for each survey wave *t*, we examined the cross-sectional association between working hours and concurrent smoking status in wave *t* while controlling for the confounders in wave *t*. Second, for smoking initiation analysis, we examined the prospective association between working hours in wave *t* and smoking initiation in wave *t+1* while controlling for the confounders in wave *t*. We included observations in which the survey participants reported they did not smoke in wave *t*, excluding observations in which the survey participants reported they already smoked in wave *t*. Third, for smoking cessation analysis, we examined the prospective association between working hours in wave *t* and smoking cessation in in wave *t+1* while controlling for the confounders in wave *t*. We included observations in which the survey participants reported that they smoked in wave *t*, excluding observations in which they reported they did not smoke in wave *t*. We employed GEE models with exchangeable working correlation matrix and logit link function. Effect sizes were presented as odds ratios (OR) and their corresponding 95% confidence intervals (CI).

As a next step, we employed a meta-analysis to combine the estimates for the associations between working hours and each outcome derived from each cohort. A random effect model was employed for meta-analysis. We evaluated the heterogeneity using Cochran’s Q test and quantified it using I^2^ statistics. Subgroup analyses were conducted to explore whether the association between long working hours and each outcome differs by worker’s characteristics including gender, age group, socio-economic status (including education and income levels), and occupational types. Following the methodology employed in previous meta-analyses (2, 3, 19), subgroup analyses explored the differential association of exposure to working ≥55 hours/week and outcomes in comparison to 35–40 hours/week across subgroups.

Several additional analyses were performed. First, we conducted an additional subgroup analysis to explore whether the association between long working hours and subsequent changes in smoking behaviors differs according to past smoking habits. Specifically, we explored whether the association between working ≥55 hours/week and smoking initiation differs between never and former smokers. Then, we explored whether the association between working ≥55 hours/week and smoking initiation differs by cessation time. We also explored whether the association between working ≥55 hours/week and smoking cessation differs by the duration of smoking among current smokers. Second, we excluded individuals who reported subjective health deterioration during the follow-up in the analysis of smoking cessation because it might serve as another significant motivation to quit smoking. Subjective health deterioration was defined as individuals who responded to the question about their health status as having declined from either “neutral,” “good,” or “very good” to the categories of “poor” or “very poor.” Third, we explored whether changes in working hour between two consecutive survey waves are associated with initiation or cessation of smoking. Specifically, we categorized change in working hour in wave *t* and wave *t+1* as <55 → <55 (reference), <55 → ≥55, ≥55 → <55, ≥55 → ≥55 hours and explored whether consecutive long working hours or increase in working hours is associated with smoking initiation or cessation in wave *t+1*. Fourth, we employed Cox proportional hazards models to explore how working hour at the baseline is associated with the smoking initiation or cessation during the follow-up among smokers and non-smokers, respectively. Fifth, we employed a multiple imputation with chained equation to handle observations containing missing values on main variables, covariates, or follow-up smoking status (21). For each dataset, a total of 20 imputed datasets were generated, and estimates were combined based on the Rubin’s rule.

All statistical analyses and visualizations were conducted using R software for Windows (version 4.2.3; R Foundation for Statistical Computing, Vienna, Austria). GEE models were fitted using “*geepack*” package and meta-analyses were performed using “*meta*” package in R.

## Results

The characteristics of observations according to each database are presented in supplementary table S2. A total of 50 508 survey participants (22 881 women) provided 284 782 observations. Prevalence of cigarette smoking during the entire observation periods were 27.9% for KLIPS, 28.9% for KHPS, 25.8% for KWPS, and 22.6% for KLOSA. Among the observations, 46.4% had blue-collar occupations, 20.8% were service/sales workers, and 32.8% had white-collar occupations. Supplementary figure S2 shows the trend of smoking prevalence observed in the KLIPS cohort.

Table 1 shows the associations of working hours with current smoking and initiation and cessation of smoking in each cohort. The crude/adjusted OR (95% CI) of the association between working ≥55 hours/week and current smoking, smoking initiation, and smoking cessation are presented. In the entire cohort, the overall prevalence of current cigarette smoking was 24.4% (25 328/103 932) among individuals working 35–40 hours/week, and 34.6% (17 853/51 602) among those working ≥55 hours/week. Additionally, 3.0% (1928/64 817) initiated and 11.3% (1297/21 211) ceased smoking during follow-up among those working 35-40 hours/week, while 4.4% (1305/29 542) initiated and 11.6% (1792/15 366) ceased smoking during follow-up among those working ≥55 hours/week.

**Table 1:** Association of working hours per week with current smoking, smoking initiation, and smoking cessation in each dataset. [OR=odds ratio; CI=confidence intervals]

		Current smoking				Smoking initiation				Smoking cessation		
		Crude model		Adjusted model ^a^		Crude model		Adjusted model ^a^		Crude model		Adjusted model ^a^
		OR (95% CI)		OR (95% CI)		OR (95% CI)		OR (95% CI)		OR (95% CI)		OR (95% CI)
KLIPS (hours) ^b^												
	<35	0.95 (0.92–0.98)		0.98 (0.93–1.02)		0.62 (0.55–0.70)		0.95 (0.82–1.11)		1.04 (0.92–1.18)		0.92 (0.80–1.05)
	35–40	Reference		Reference		Reference		Reference		Reference		Reference
	41–48	1.11 (1.08–1.14)		1.03 (1.00–1.07)		1.42 (1.30–1.56)		0.96 (0.87–1.07)		1.08 (0.99–1.18)		0.88 (0.80–0.97)
	49–54	1.11 (1.07–1.15)		1.05 (1.01–1.09)		1.56 (1.39–1.75)		1.18 (1.04–1.35)		0.99 (0.90–1.10)		0.84 (0.75–0.95)
	≥55	1.20 (1.16–1.25)		1.09 (1.04–1.13)		1.80 (1.64–1.97)		1.08 (0.96–1.21)		1.15 (1.06–1.25)		0.91 (0.82–1.00)
KHPS (hours) ^c^												
	<35	0.97 (0.92–1.03)		1.16 (1.05–1.27)		0.36 (0.23–0.57)		0.65 (0.40–1.05)		0.85 (0.61–1.18)		0.82 (0.57–1.19)
	35–40	Reference		Reference		Reference		Reference		Reference		Reference
	41–48	1.09 (1.03–1.14)		1.07 (1.00–1.15)		0.96 (0.73–1.26)		0.86 (0.64–1.14)		0.95 (0.75–1.20)		1.02 (0.80–1.30)
	49–54	1.12 (1.06–1.19)		1.09 (1.01–1.17)		1.29 (0.96–1.74)		0.91 (0.66–1.24)		0.90 (0.69–1.16)		1.00 (0.76–1.30)
	≥55	1.15 (1.09–1.22)		1.07 (1.00–1.15)		1.34 (1.03–1.75)		0.85 (0.64–1.13)		0.88 (0.71–1.11)		1.01 (0.80–1.28)
KWPS (hours) ^d^												
	<35	1.01 (0.98–1.04)		0.97 (0.93–1.01)		0.80 (0.71–0.90)		0.97 (0.85–1.11)		1.02 (0.91–1.14)		0.97 (0.84–1.11)
	35–40	Reference		Reference		Reference		Reference		Reference		Reference
	41–48	1.06 (1.03–1.09)		1.01 (0.98–1.04)		0.95 (0.84–1.07)		0.85 (0.75–0.97)		0.82 (0.72–0.93)		0.84 (0.73–0.96)
	49–54	1.09 (1.05–1.13)		1.02 (0.98–1.06)		1.32 (1.16–1.52)		1.06 (0.92–1.22)		0.83 (0.72–0.95)		0.87 (0.75–1.01)
	≥55	1.17 (1.13–1.21)		1.06 (1.02–1.10)		1.30 (1.14–1.48)		1.00 (0.87–1.15)		0.83 (0.73–0.93)		0.88 (0.77–1.00)
KLOSA (hours) ^e^												
	<35	0.92 (0.86–0.98)		0.97 (0.90–1.06)		0.92 (0.66–1.29)		1.48 (1.02–2.17)		0.91 (0.72–1.15)		0.92 (0.71–1.17)
	35–40	Reference		Reference		Reference		Reference		Reference		Reference
	41–48	1.20 (1.12–1.29)		1.11 (1.03–1.21)		1.43 (1.03–2.00)		1.23 (0.85–1.76)		0.94 (0.74–1.20)		1.00 (0.78–1.27)
	49–54	1.27 (1.17–1.37)		1.13 (1.04–1.24)		1.38 (0.94–2.02)		1.14 (0.75–1.73)		0.87 (0.67–1.14)		0.95 (0.72–1.24)
	≥55	1.28 (1.20–1.37)		1.07 (1.00–1.16)		1.13 (0.83–1.54)		0.98 (0.69–1.39)		0.86 (0.70–1.06)		0.93 (0.75–1.16)

^a^ Adjusted model controlled for gender, age, education level, income level, marital status, occupation type, and survey year ^b^ N (Observations) – current smoking: 21 206 (145 770); smoking initiation: 16 180 (89 578); smoking cessation: 6407 (35 465). ^c^ N (Observations) – current smoking: 7900 (18 912); smoking initiation: 5483 (12 665); smoking cessation: 2269 (5050). ^d^ N (Observations) – current smoking: 15 624 (96 397); smoking initiation: 10 005 (56 955); smoking cessation: 3653 (19 122). ^e^ N (Observations) – current smoking: 5778 (23 703); smoking initiation: 4384 (15 452); smoking cessation: 1482 (4618).

**Figure 1:**
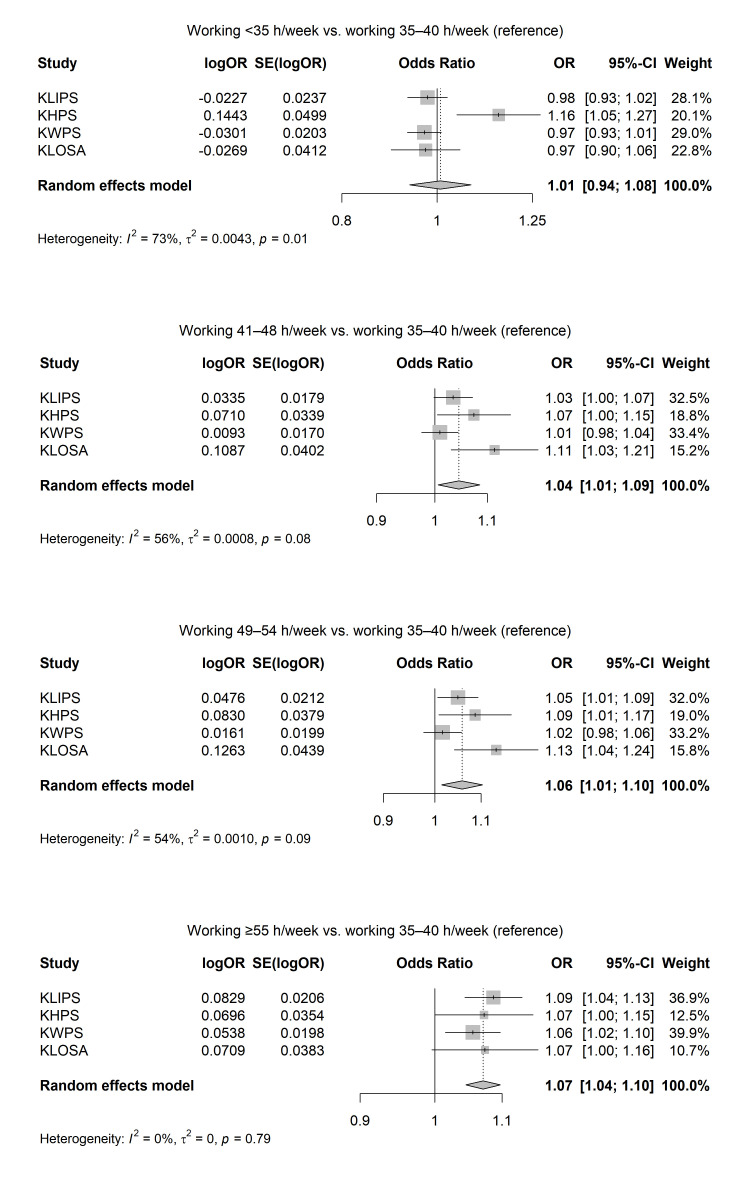
Results of random-effect meta-analyses on the associations between long working hours and current smoking among Korean workers, adjusted for gender, age, education level, income level, marital status, occupation type, and survey year.

Figure 1 presents the results of the meta-analysis on the association between working hours per week and current smoking. The adjusted OR of the association between working hours per week and current smoking were 1.01 (95% CI 0.94–1.08) for <35 hours/week (I^2^=73%; P for the heterogeneity=0.01), 1.04 (95% CI 1.01–1.09) for 41–48 hours/week (I^2^=56%; P for the heterogeneity=0.08), 1.06 (95% CI 1.01–1.10) for 49–54 hours/week (I^2^=54%; P for the heterogeneity=0.09), and 1.07 (95% CI 1.04–1.10) for ≥55 hours/week (I^2^=0%; P for the heterogeneity=0.79).

**Figure 2:**
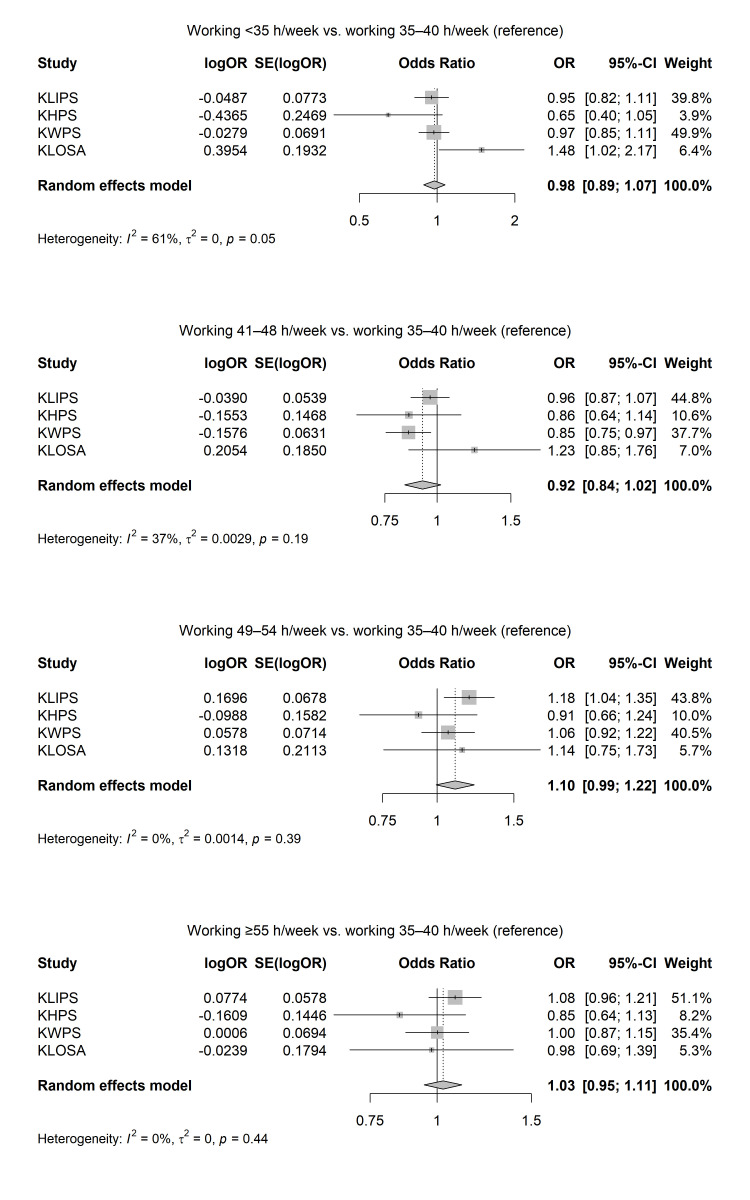
Results of random-effect meta-analyses on the associations between long working hours and smoking initiation among Korean workers, adjusted for gender, age, education level, income level, marital status, occupation type, and survey year.

Figure 2 presents the results of the meta-analysis on the association between working hours per week and smoking initiation after follow-up. The adjusted OR of the association between working hours per with and smoking initiation were 0.98 (95% CI 0.89–1.07) for <35 hours/week (I^2^=61%; P for the heterogeneity=0.05), 0.92 (0.84–1.02) for 41–48 hours/week (I^2^=37%; P for the heterogeneity=0.19), 1.10 (95% CI 0.99–1.22) for 49–54 hours/week (I^2^=0%; P for the heterogeneity=0.39), and 1.03 (95% CI 0.95–1.11) for ≥55 hours/week (I^2^=0%; P for the heterogeneity=0.44).

**Figure 3:**
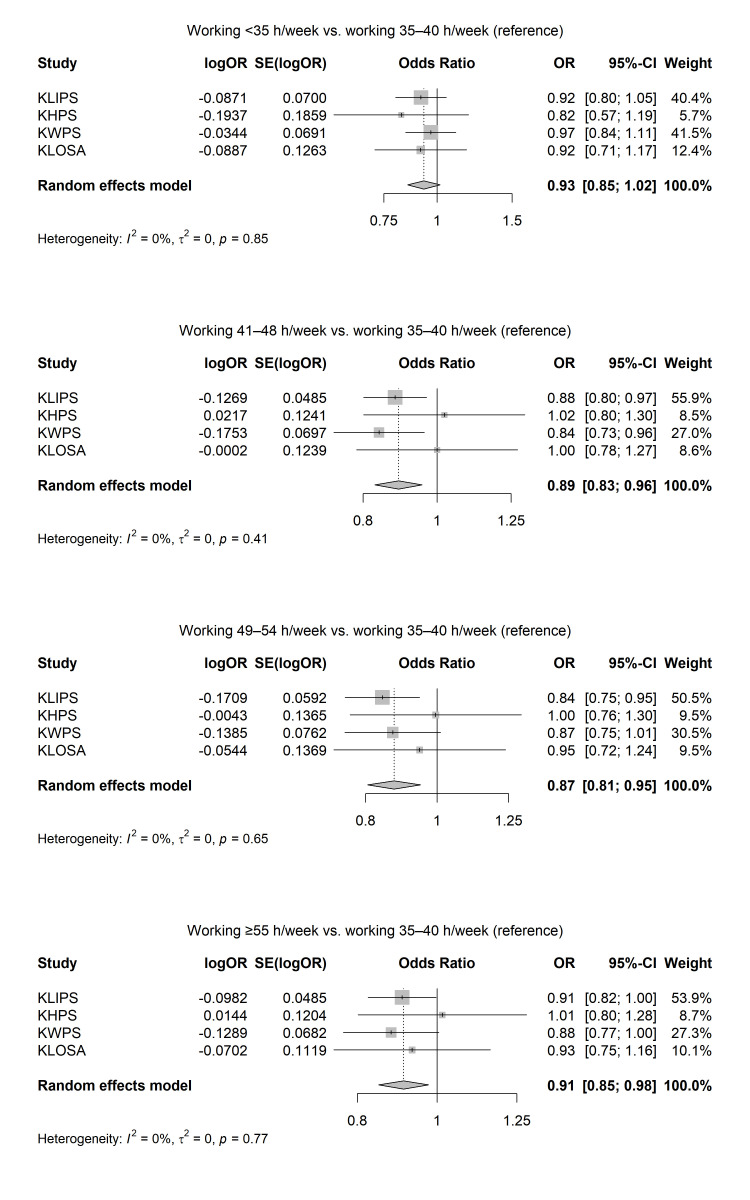
Results of random-effect meta-analyses on the associations between long working hours and smoking cessation among Korean workers, adjusted for gender, age, education level, income level, marital status, occupation type, and survey year.

Figure 3 presents the results of the meta-analysis on the association between working hours per week and smoking cessation after follow-up. The adjusted OR of the association between working hours per with and smoking cessation were 0.93 (95% CI 0.85–1.02) for <35 hours/week (I^2^=0%; P for the heterogeneity=0.85), 0.89 (0.83–0.96) for 41–48 hours/week (I^2^=0%; P for the heterogeneity=0.41), 0.87 (95% CI 0.81–0.95) for 48–54 hours/week (I^2^=0%; P for the heterogeneity=0.65), and 0.91 (95% CI 0.85–0.98) for ≥55 hours/week (I^2^=0%; P for the heterogeneity=0.77).

**Figure 4:**
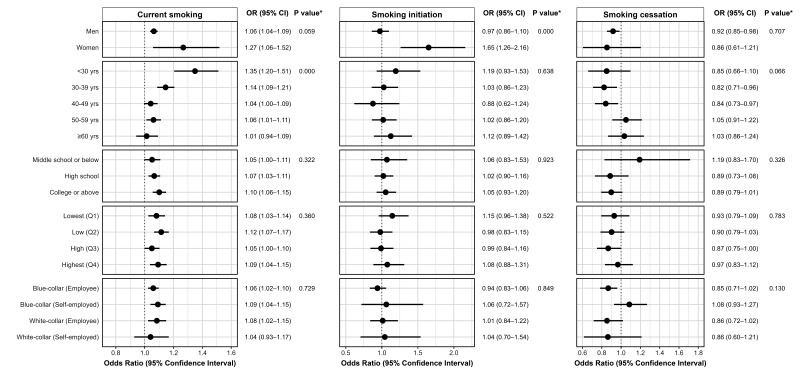
Subgroup analysis on the association between working ≥55 hours per week and current smoking, smoking initiation, and smoking cessation by gender, age, socio-economic status, and occupational types (OR=odds ratio; CI=confidence intervals).

Figure 4 shows the results of subgroup analyses. The association between ≥55 hours/week and current smoking was pronounced among young-aged workers (P for the subgroup difference <0.001). Additionally, the association between ≥55 hours/week and smoking initiation at the follow-up was pronounced among women than men (P for the subgroup difference <0.001).

Supplementary figures S3–S5 indicate that the relationship between long working hours and smoking initiation or cessation did not show clear variations based on past smoking history. Supplementary table S3 shows that individuals exposed to consecutive long working hours exhibited a reduced likelihood of smoking cessation in comparison to those whose weekly working hours consistently remained <55 hours. Supplementary figure S6 shows that the association between long working hours and smoking cessation was maintained after excluding those experiencing subjective health deterioration. Supplementary table S4 shows that the association between long working hours and smoking cessation was mitigated on Cox regression models with wider confidence intervals. Finally, results from the imputed dataset produced comparable results with those in main analyses (supplementary figure S7-S9).

## Discussion

In our study, we observed that Korean workers working >40 hours/week, in comparison to those working 35–40 hours/week, were associated with a higher OR of current smoking and a reduced likelihood of smoking cessation. Additionally, the effect sizes were similar across the categories of 41–48, 49–54, and ≥55 hours/week. Conversely, no clear associations between working hours and smoking initiation were observed in this study. Those working short hours (<35 hours/week) also did not have clear association with smoking behaviors. Although the effect sizes of long working hours on smoking behaviors were observed to be modest, our findings suggest that those working long hours may be less likely to quit smoking. This underscores the necessity of policy interventions to reduce excess working hours and promote smoking cessation among workers subjected to long working hours.

Consistent with prior studies indicating a declining trend in smoking prevalence in Korea, the overall prevalence of cigarette smoking demonstrated a gradual decrease throughout the survey period in this study (22, 23). This decline may be attributed to the effectiveness of ongoing anti-smoking campaigns and legislative measures implemented in Korea over the past years (24). However, despite these initiatives, the reduction in smoking prevalence is particularly notable among males, whereas females exhibit a stagnation. Moreover, smoking prevalence was observed to be higher among middle-aged workers and individuals with blue-collar occupations, which aligns with that observed in the general Korean population (22, 23). Consequently, this underscores the need for future anti-smoking policies to focus on these demographics.

Our results are consistent with previous findings that long working hours are associated with a concurrent cigarette smoking (10–13). Additionally, our findings are in line with previous studies that long working hours are associated with reduced likelihood of smoking cessation. For instance, a previous Korean study suggested that working >52 hours/week reduced workers’ intention to quit smoking (11). Similarly, Angrave et al (10) demonstrated that working >60 hours/week reduced the chance of smoking cessation. Our study contributes to the body of literature by demonstrating that long working hours are not only linked to a higher prevalence of current smoking but also to a reduced likelihood of smoking cessation among Korean workers within the longitudinal framework.

Long working hours are a major cause of increased job stress, which can induce workers to engage in addictive behaviors. Workers subjected to long working hours may adopt unhealthy lifestyles to relieve their job-related stress (15). For example, previous studies have found that high job stress is associated with alcohol consumption and internet addiction (7, 25, 26), and that job stress has been found to increase the likelihood of smoking initiation and reduce the likelihood of smoking cessation (27). High job stress is also found to increase nicotine dependence and smoking risk (28). According to previous studies, chronic stress changes the hormonal systems (hypothalamic–pituitary–adrenocortical axis) to react more sensitively to nicotine exposure, leading to an increased risk of smoking (29–31). Consequently, long working hours may inhibit workers from quitting smoking by causing chronic stress. Unlike current smoking and smoking cessation, the association between long working hours and smoking initiation was inconclusive in this study. One potential explanation for the OR for smoking initiation being not pronounced among those working ≥55 hours/week is the likelihood that exposure to the extreme category of long working hours can induce great time pressure. This, in turn, may result in workers having insufficient break time for smoking or diverting their attention away from the intention to initiate smoking.

In the subgroup analysis, long working hours are associated with an increased likelihood of smoking initiation in the following wave among female workers. The pronounced association observed among women may be related to the double burden that women bear. In Korean society, female workers are expected to bear a substantial burden of family care roles alongside their professional responsibilities (32). This circumstance can intensify the impact of time constraints resulting from long working hours, potentially leading to increased stress and the initiation of smoking (33, 34). Additionally, the subgroup analysis shows that those exposed to long working hours are more likely to engage in cigarette smoking, especially young-aged workers. While the underlying mechanism remains unclear, young workers with long working hours may also simultaneously face other precarious employment conditions (35). For instance, gig workers, prevalent among the young workforce in Korea, are often exposed to overwork, along with employment instability, lack of rights and protection, and exposure to psychological hazards (36). Consequently, the heightened stress level could potentially increase the likelihood of engagement in cigarette smoking. Our subgroup analyses indicate that the relationship between long working hours and smoking behaviors may vary depending on the demographic characteristics of workers. This variability could be one of the reasons for differences in observed estimates across cohorts. Therefore, conducting targeted epidemiological studies in the future, specifically focusing on the impact of long working hours on smoking behavior among young or female workers, would contribute to a more comprehensive understanding.

Our study has several policy implications. The findings can be viewed as supporting the current theoretical perspective, suggesting that unhealthy lifestyles may serve as mediators in the causal link between long working hours and adverse health outcomes, including cardiovascular diseases and mortality (2, 3). Furthermore, a recent study conducted in China revealed that the impact of long working hours on all-cause mortality may be more pronounced among smokers than non-smokers (37). Consequently, proactive interventions that promote smoking cessation could prove beneficial in mitigating health risks for workers subjected to long working hours and overwork. Additionally, Ahn demonstrated that a reduction in working hours leads to a decreased likelihood of smoking (38). Therefore, reducing working hours, in addition to implementing a direct anti-smoking policy, can be a key strategy to induce workers to quit smoking.

The following limitations of this study should be noted. First, owing to the observational nature of our study design, we could not assert the causal effect of long working hours on workers’ smoking behaviors. Second, the social desirability bias should be considered. In other words, smokers may be reluctant to report their smoking status, as smoking is still regarded as a social taboo, for both men and women in the Asian context. A previous study reported that the cotinine-verified smoking rate was higher than self-reported smoking rates (39). In future studies, the relationship between working hours and smoking should be identified using objective measurements such as urine cotinine. Third, working hours were also assessed using self-reported questionnaire, leading to potential measurement errors, including recall bias. Fourth, multidimensional stress factors induced by long working hours, such as job stress and work-family conflict, were not considered due to a lack of data. Fifth, our findings may not necessarily apply to other nations with different work cultures and organizational policies. The health effects of long working hours have been known to vary across countries (40). Sixth, there is a possibility that the presence of missing values leads to biased estimation, considering the GEE models assume the missing-at-completely-random condition. Nonetheless, as confirmed in the aforementioned sensitivity analysis (supplementary figures S1-S3), the results derived from imputed datasets also yielded similar outcomes. Seventh, the Cox regression did not yield a clear association between working hours and cessation. The disparity in results may be attributed to the fact that GEE analysis, compared with Cox model, focused on the short-term effects of long working hours, while also allowing for the consideration for the multiple events during the study periods. Eighth, while we adjusted for the calendar effect, it may not fully account for the broader shifts in the labor market. For instance, the recent expansion of the gig economy might exert more pronounced effects on working hours and employment conditions among younger workforce (35). Indeed, our stratified analysis shows that the association between long working hours and smoking risk is pronounced among young-aged workers. The lack of information on the dynamics of precarious employment across demographics limited in-depth analyses in this study, necessitating the need for more comprehensive investigations in future.

### Concluding remarks

The key finding of our research is that those working long hours (>40 hours/week) were related to an increased OR of current smoking and a decreased likelihood of smoking cessation among Korean workers. This implies that the heightened smoking risk among workers may be one of the contributing factors to the health deterioration associated with long working hours. Consequently, implementing effective health promotion initiatives aimed at encouraging individuals with long working hours to quit smoking and improving their overworked conditions is imperative.

## Supplementary material

Supplementary material
