# Supplementary material for: Relationship between long working hours and smoking behaviors: Evidence from population-based cohort studies in Korea
Source: Scand J Work Environ Health. 2024 Apr 29;50(4):257–67. doi: 10.5271/sjweh.4147 (PMC11129822; doi:10.5271/sjweh.4147)
Supplement: Supplementary material [file SJWEH-50-257-S001.pdf]

Relationship between long working hours and smoking behaviors: Evidence from population-based cohort studies in Korea[1]

by Seong-Uk Baek, MD, Myeong-Hun Lim, MD, Taeyeon Kim, MD, Yu-Min Lee, PhD, Jong-Uk Won, PhD, Jin-Ha Yoon, PhD 2

1. *Supplementary material*
2. *Correspondence to: Jin-Ha Yoon, MD, PhD, Department of Preventive Medicine, Yonsei University College of Medicine, Yonsei University Health System, 50-1 Yonsei-ro, Seodaemun-gu, Seoul 03722, Republic of Korea. [E-mail: seonguk3411@gmail.com]*

Table S1. Characteristics of included datasets in Korea for analysis of long working hours and smoking behaviors.

Datasets	Current smoking analysis		Smoking initiation analysis		Smoking cessation analysis		Exposure assessment (Weekly working hours)	Outcome assessment (Smoking status)	Study period (Waves)
	N of study participants	N of observations	N of study participants	N of observations	N of study participants	N of observations			
KLIPS	21,206	145,770	16,180	89,578	6407	35,465	What is the average number of working hours per week?	Do you usually smoke cigarettes?	2005–2021 WAVE 8–24
KHPS	7900	18,912	5483	12,665	2269	5050	What is the average number of working hours per week?	Do you currently smoke cigarettes?	2011–2014 WAVE 4–7
KWPS	15,624	96,397	56,955	10,005	3653	19,122	How many hours do you work per week in your job?	Do you currently smoke cigarettes?	2008–2022 WAVE 3–17
KLOSA	5778	23,703	4384	15,452	1482	4618	What is the average number of working hours per week?	Do you currently smoke cigarettes?	2006–2020 WAVE 1–8

KLIPS: Korean Labor & Income Panel Study; KOWEPS: Korea Welfare Panel Study; KHPS: Korea Health Panel Study; KLOSA: Korean Longitudinal Study of Aging

Table S2. Socio-demographic characteristics of survey participants from each dataset.

	Total	KLIPS	KHPS	KWPS	KLOSA
**Gender**					
Men	166,947 (58.6)	85,659 (58.8)	10,835 (57.3)	56,317 (58.4)	14,136 (59.6)
Women	117,835 (41.4)	60,111 (41.2)	8077 (42.7)	40,080 (41.6)	9,567 (40.4)
**Age**					
<30	28,108 (9.9)	14,403 (9.9)	2191 (11.6)	11,514 (11.9)	0 (0.0)
30–39	55,564 (19.5)	32,330 (22.2)	4392 (23.2)	18,842 (19.5)	0 (0.0)
40–49	69,453 (24.4)	38,191 (26.2)	5782 (30.6)	23,589 (24.5)	1891 (8.0)
50–59	65,330 (22.9)	31,962 (21.9)	3916 (20.7)	18,743 (19.4)	10,709 (45.2)
≥60	66,327 (23.3)	28,884 (19.8)	2631 (13.9)	23,709 (24.6)	11,103 (46.8)
**Education level**					
Middle school or below	72,562 (25.5)	33,451 (22.9)	3313 (17.5)	25,013 (25.9)	10,785 (45.5)
High school	103,846 (36.5)	54,391 (37.3)	7365 (38.9)	32,907 (34.1)	9183 (38.7)
College or above	108,374 (38.1)	57,928 (39.7)	8234 (43.5)	38,477 (39.9)	3735 (15.8)
**Income level**					
Q1	71,429 (25.1)	36,642 (25.1)	4680 (24.7)	24,111 (25.0)	5996 (25.3)
Q2	71,566 (25.1)	36,464 (25.0)	4792 (25.3)	24,101 (25.0)	6209 (26.2)
Q3	71,132 (25.0)	36,269 (24.9)	4784 (25.3)	24,097 (25.0)	5982 (25.2)
Q4	70,655 (24.8)	36,395 (25.0)	4656 (24.6)	24,088 (25.0)	5516 (23.3)
**Marital status**					
Married	206,133 (72.4)	106,017 (72.7)	13,473 (71.2)	65,888 (68.4)	20,755 (87.6)
Unmarried	50,068 (17.6)	25,335 (17.4)	4047 (21.4)	17,957 (18.6)	2729 (11.5)
Others	28,581 (10.0)	14,418 (9.9)	1392 (7.4)	12,552 (13.0)	219 (0.9)
**Occupation type**					
Blue collar (employee)	120,166 (42.2)	57,613 (39.5)	11,396 (60.3)	42,375 (44.0)	8782 (37.1)
Blue collar (employer)	71,120 (25.0)	36,612 (25.1)	0 (0.0)	23,949 (24.8)	10559 (44.5)
White collar (employee)	81,587 (28.6)	44,036 (30.2)	7516 (39.7)	27,332 (28.4)	2703 (11.4)
White collar (employer)	11,909 (4.2)	7509 (5.2)	0 (0.0)	2741 (2.8)	1659 (7.0)
**Smoking status**					
Yes	76,412 (26.8)	40,695 (27.9)	5457 (28.9)	24,906 (25.8)	5354 (22.6)
No	208,370 (73.2)	105,075 (72.1)	13,455 (71.1)	71,491 (74.2)	18,349 (77.4)
**N of survey participations**	284,782	21,206	7900	15,624	5778
**N of observations**	50,508	145,770	18,912	96,397	23,703
**Survey year (WAVE)**		2005–2021 (WAVE 8–24)	2011–2014 (WAVE 4–7)	2008–2022 (WAVE 3–17)	2006–2020 (WAVE 1–8)

KLIPS: Korean Labor & Income Panel Study; KWPS: Korea Welfare Panel Study; KHPS: Korea Health Panel Study; KLOSA: Korean Longitudinal Study of Aging

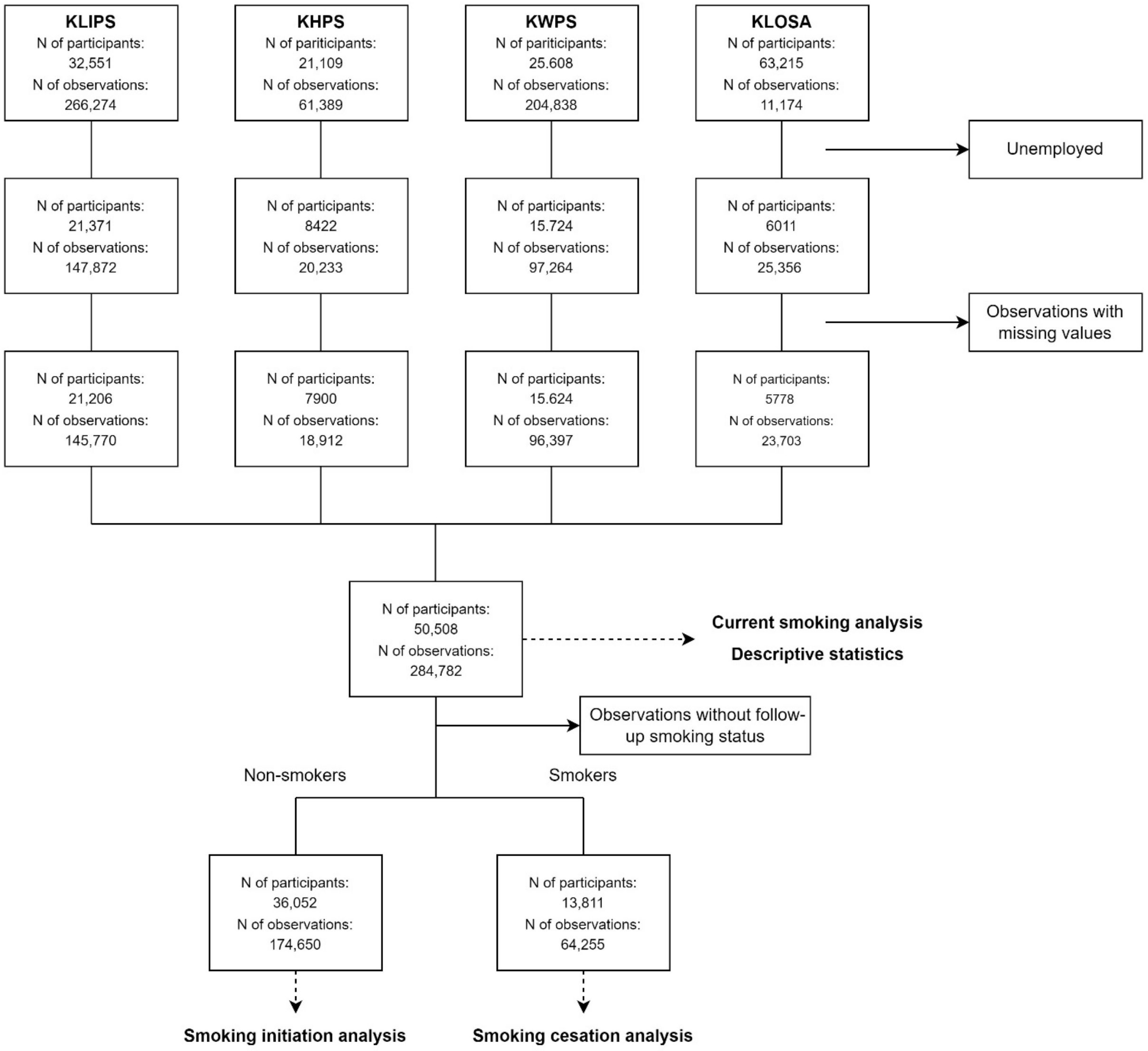

Figure S1 Flowchart of the selection of survey participants

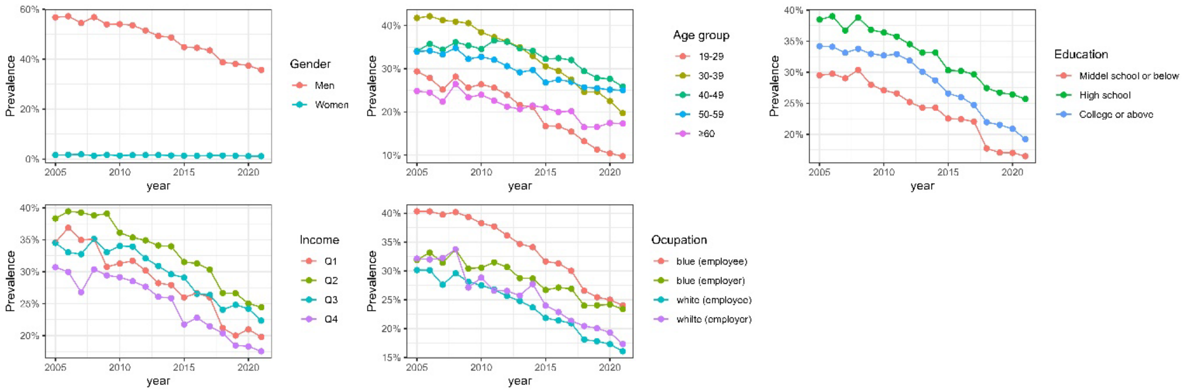

Figure S2 Overall trend of smoking prevalence during study period observed in the KLIPS cohort.

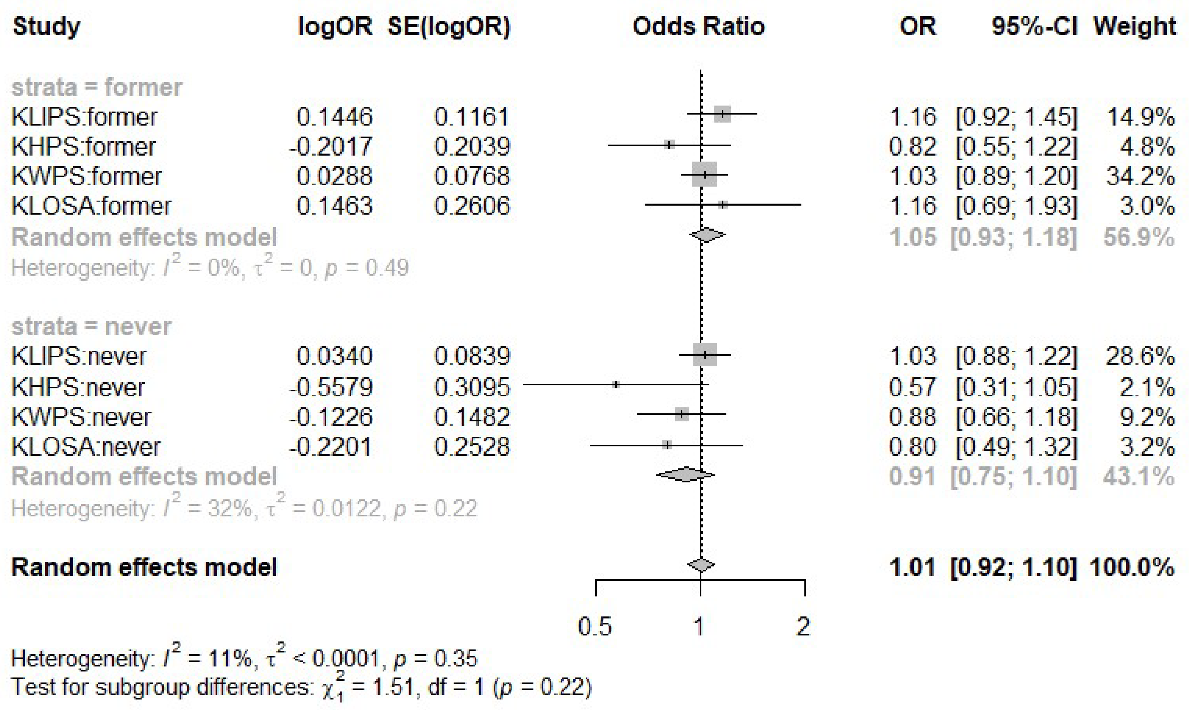

Figure S3 Association between working ≥55 h per week and smoking initiation in the following wave, stratified by the past smoking history (former smokers vs. never smokers) (OR, odds ratio; CI, confidence interval).

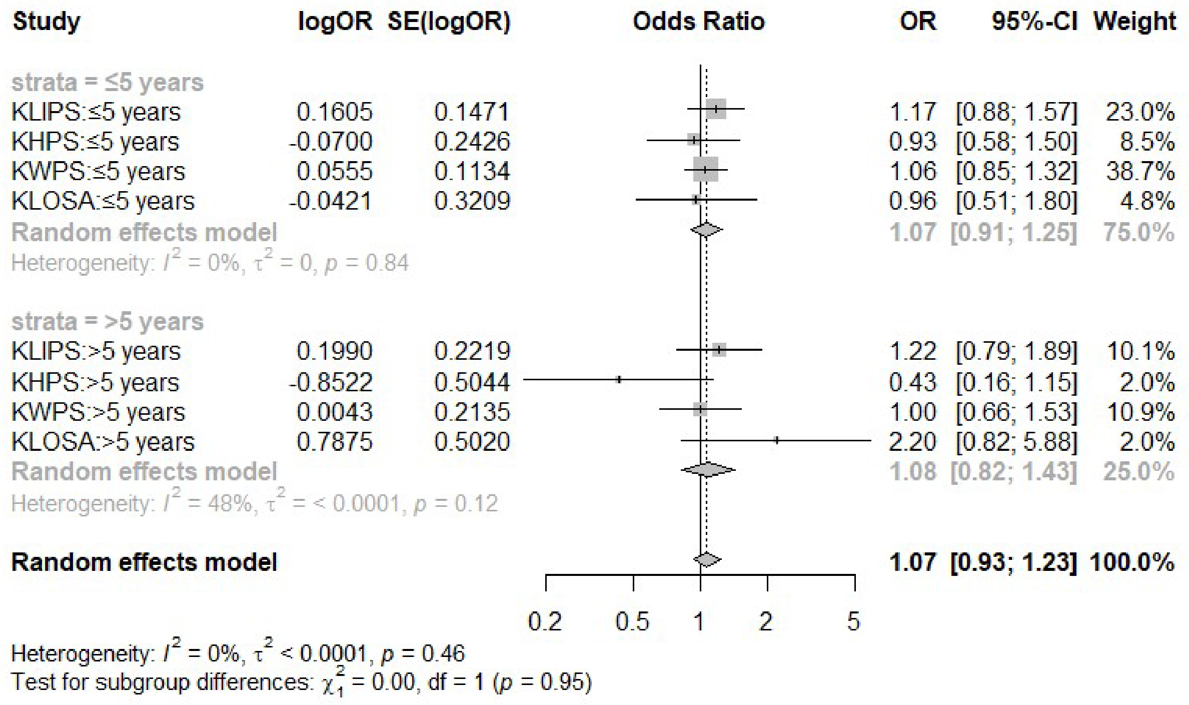

Figure S4 Association between working ≥55 h per week and smoking initiation in the following wave among former smokers, stratified by the duration of cessation (≤5 years vs. >5 years) (OR, odds ratio; CI, confidence interval).

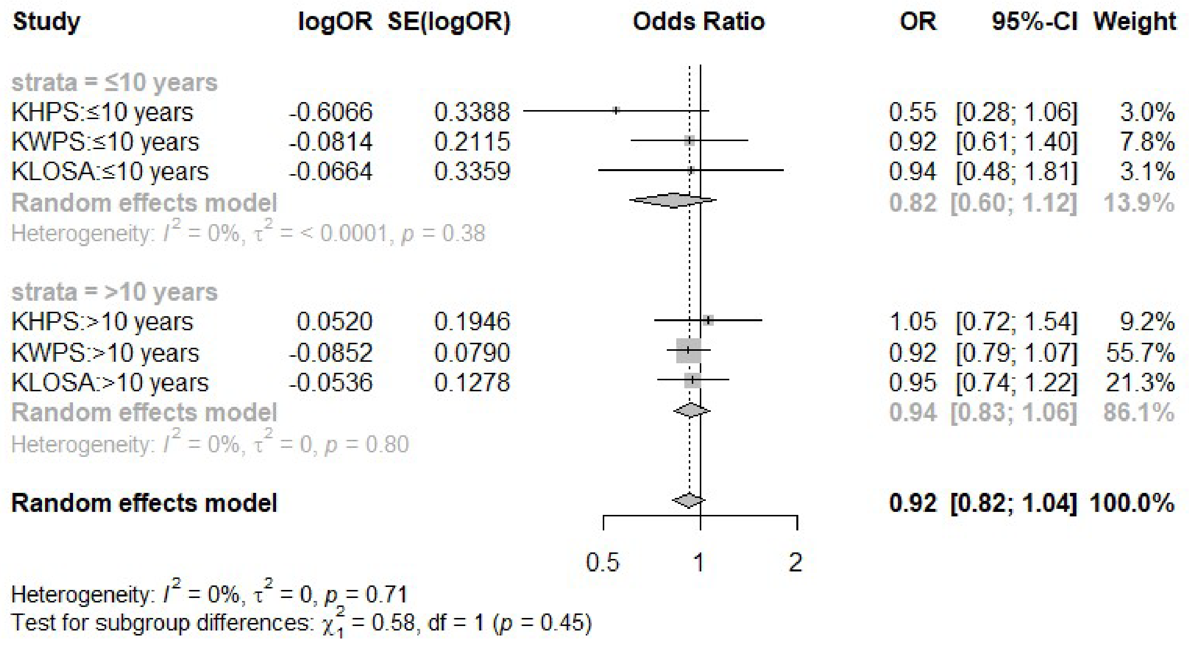

Figure S5 Association between working ≥55 h per week and smoking cessation in the following wave among smokers, stratified by the duration of smoking (≤10 years vs. >10 years). KLIPS cohort was excluded due to lack of information on the duration of smoking. (OR, odds ratio; CI, confidence interval).

Table S3 Association between changes in working hours and initiation or cessation of smoking

	**Smoking initiation (in Wave *t+1*)**	**Smoking cessation (in Wave *t+1*)**
	**OR (95% CI)**	**OR (95% CI)**
**Changes in working hour**		
(Wave *t* → Wave *t+1*)		
<55 h → <55 h	1.00 (1.00–1.00)	1.00 (1.00–1.00)
<55 h → ≥55 h	1.12 (0.89–1.41)	0.94 (0.87–1.02)
<55 h → <55 h	1.05 (0.91–1.20)	1.04 (0.97–1.12)
<55 h → <55 h	1.02 (0.86–1.20)	0.90 (0.84–0.98)

OR, odds ratio; CI, confidence interval.

Estimates were drawn from the random-effect meta-analyses.

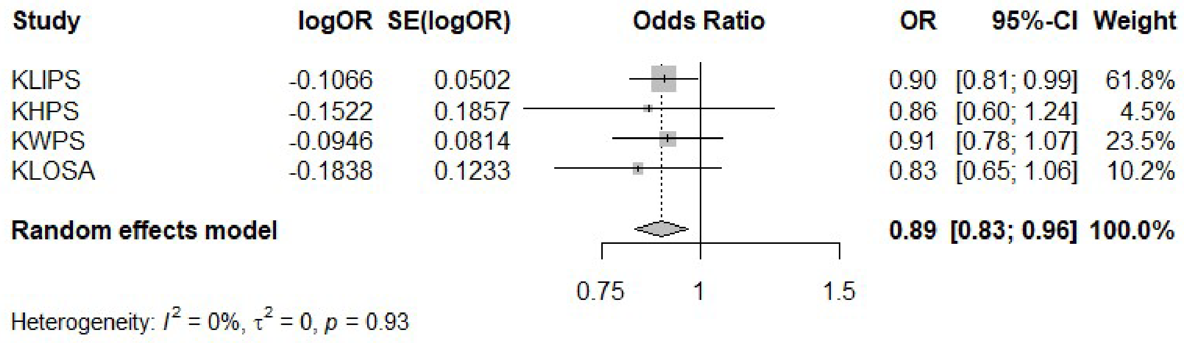

Figure S6 Association between working ≥55 h per week and smoking cessation in the following wave, excluding those who experienced subjective health deterioration during the follow-up (OR, odds ratio; CI, confidence interval).

Table S4 Association between working hours and initiation or cessation of smoking on Cox regression models

	Smoking initiation	Smoking cessation
	**HR (95% CI)**	**HR (95% CI)**
**Working hours per week**		
<35 h	0.86 (0.68–1.07)	0.96 (0.82–1.12)
35–40 h	1.00 (1.00–1.00)	1.00 (1.00–1.00)
41–48 h	0.88 (0.75–1.02)	1.07 (0.94–1.22)
49–54 h	1.00 (0.83–1.21)	0.97 (0.81–1.16)
≥55 h	0.90 (0.77–1.04)	0.94 (0.84–1.06)

HR, hazard ratio; CI, confidence interval.

Estimates were drawn from the random-effect meta-analyses.

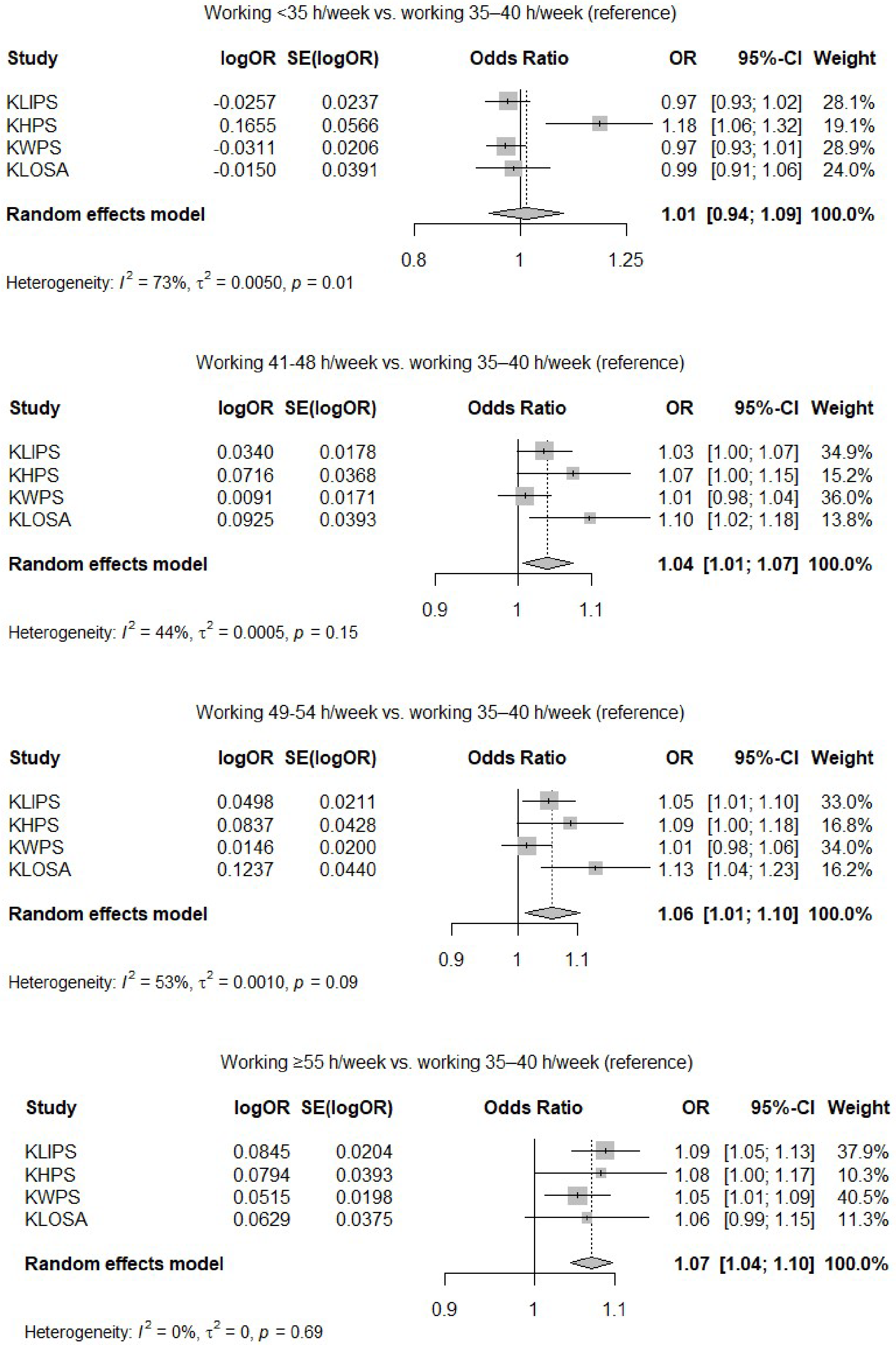

Figure S7 Results of random-effect meta-analyses on the associations between working hours and current smoking among Korean workers based on imputed datasets

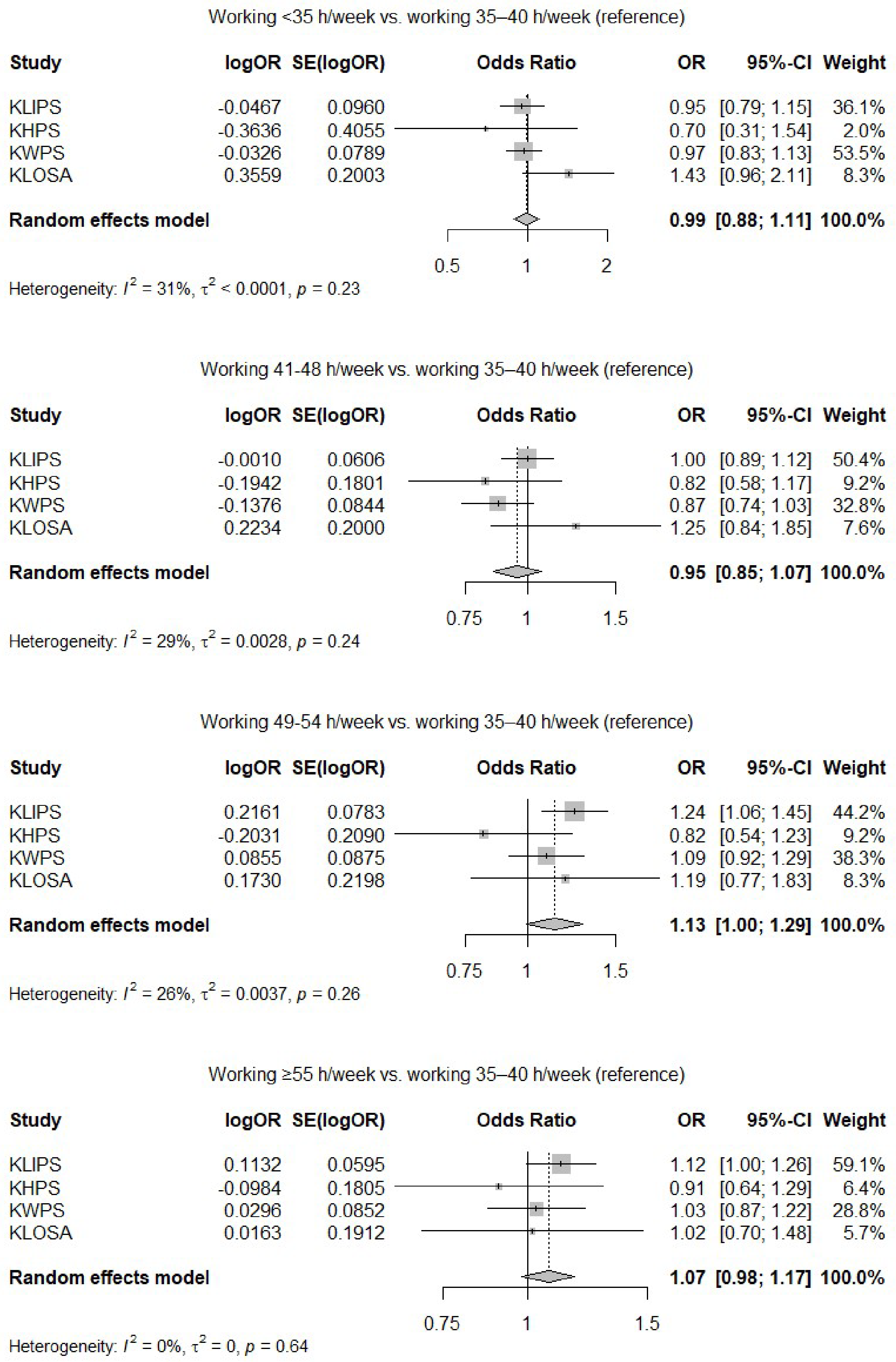

Figure S8 Results of random-effect meta-analyses on the associations between working hours and smoking initiation among Korean workers based on imputed datasets

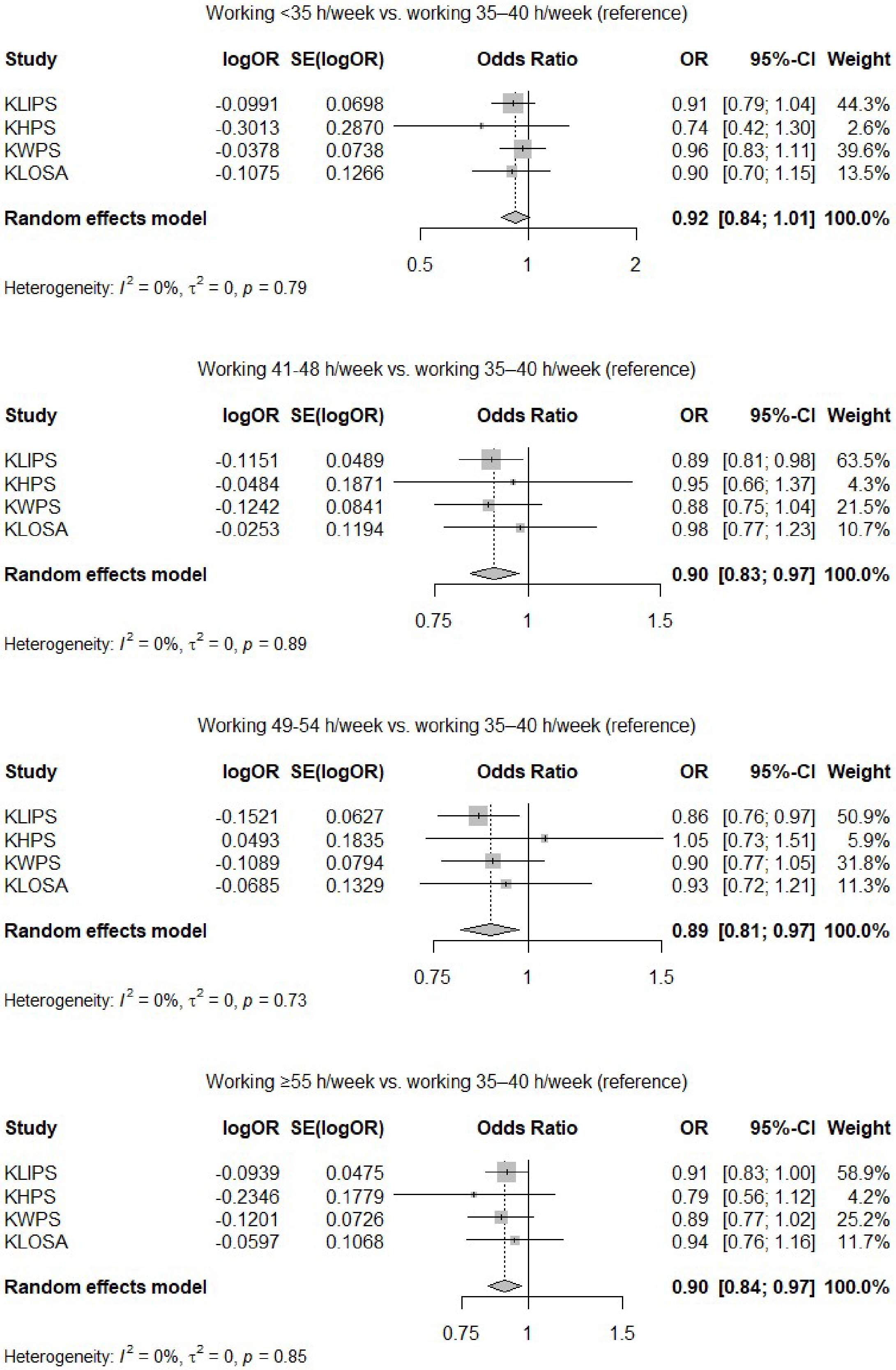

Figure S9 Results of random-effect meta-analyses on the associations between working hours and smoking cessation among Korean workers based on imputed dataset
